# A Glimpse of Inflammation and Anti-Inflammation Therapy in Diabetic Kidney Disease

**DOI:** 10.3389/fphys.2022.909569

**Published:** 2022-07-06

**Authors:** Chongbin Liu, Ming Yang, Li Li, Shilu Luo, Jinfei Yang, Chenrui Li, Huafeng Liu, Lin Sun

**Affiliations:** ^1^ Department of Nephrology, The Second Xiangya Hospital, Central South Unibersity, Changsha, China; ^2^ Hunan Key Laboratory of kidney Disease and Blood Purification, Changsha, China; ^3^ Guangdong Provincial Key Laboratory of Autophagy and Major Chronic Non-communicable Diseases & Institute of Nephrology, Affiliated Hospital of Guangdong Medical University, Zhanjiang, China,

**Keywords:** diabetic kidney disease, inflammation, signaling pathway, anti-Inflammation, anti-hyperglycemic, mineralocorticoid receptor antagonists, traditional Chinese medicine

## Abstract

Diabetic kidney disease (DKD) is a common complication of diabetes mellitus and a major cause of end-stage kidney disease (ESKD). The pathogenesis of DKD is very complex and not completely understood. Recently, accumulated evidence from *in vitro* and *in vivo* studies has demonstrated that inflammation plays an important role in the pathogenesis and the development of DKD. It has been well known that a variety of pro-inflammatory cytokines and related signaling pathways are involved in the procession of DKD. Additionally, some anti-hyperglycemic agents and mineralocorticoid receptor antagonists (MRAs) that are effective in alleviating the progression of DKD have anti-inflammatory properties, which might have beneficial effects on delaying the progression of DKD. However, there is currently a lack of systematic overviews. In this review, we focus on the novel pro-inflammatory signaling pathways in the development of DKD, including the nuclear factor kappa B (NF-κB) signaling pathway, toll-like receptors (TLRs) and myeloid differentiation primary response 88 (TLRs/MyD88) signaling pathway, adenosine 5′-monophosphate-activated protein kinase (AMPK) signaling pathways, inflammasome activation, mitochondrial DNA (mtDNA) release as well as hypoxia-inducible factor-1(HIF-1) signaling pathway. We also discuss the related anti-inflammation mechanisms of metformin, finerenone, sodium-dependent glucose transporters 2 (SGLT2) inhibitors, Dipeptidyl peptidase-4 (DPP-4) inhibitors, Glucagon-like peptide-1 (GLP-1) receptor agonist and traditional Chinese medicines (TCM).

## Introduction

Diabetic kidney disease (DKD), which is also known as diabetic nephropathy before, is the most common microvascular complication of diabetes mellitus (Thomas et al., 2015; Doshi and Friedman, 2017; Sun et al., 2022). The number of DKD patients has increased year by year, which has led to a huge burden on global public health. Recent studies show that 21.8%–40% of diabetic patients may progress to DKD, which is the main cause of end-stage renal disease (ESRD) (Zhang J. et al., 2020; Mottl et al., 2022). In addition, nearly 38.8% of ESRD in 2018 was attributable to DKD in America (Johansen et al., 2021). In China and India, the proportion of ESRD in DKD patients was about 1/5 (23%) and 1/3 (31.2%), respectively (Hussain et al., 2019; Zhang L. et al., 2019). On the other hand, treatment of DKD mainly includes lifestyle interventions, such as exercise, weight loss, smoking cessation and sodium restriction, and medication to control hyperglycemia as well as blood pressure (Doshi and Friedman, 2017; Anders et al., 2018). However, current treatments cannot completely delay the progression to ESRD. Therefore, it is still necessary to further explore the pathogenesis of DKD and find new therapeutic targets.

Previous studies demonstrate that many factors are implicated in the progression of DKD, such as metabolic abnormalities and hemodynamic changes caused by hyperglycemia and insulin resistance, etc (Forbes and Thorburn, 2018). It has been well-known that overproduction of reactive oxygen species (ROS), abnormal autophagy, senescence are also involved in the development of DKD (Ren et al., 2020; Sun et al., 2022). Recently, accumulated evidence suggested that inflammation plays a key role in the pathophysiology of DKD. For example, oxidative stress (Huang W. et al., 2020), abnormal autophagy (Han et al., 2021), and senescence (Xiong and Zhou, 2019) could lead to the abnormal immune-inflammation in DKD. Furthermore, several clinical trials found that non-steroidal selective mineralocorticoid receptor antagonists (MRA) could delay the progression of DKD by inhibiting inflammation (Donath, 2014; Komada and Muruve, 2019). These data strongly suggest that inflammation plays an important role in the development of DKD.

Inflammation is a biologically conserved immune defense mechanism (Cronkite and Strutt, 2018). In human, inflammation plays a dual role in various disease. Early inflammatory responses may promote tissue repair. However, severe inflammation would lead to secondary injury resulting in tissue damage or fibrosis (Nathan and Ding, 2010; Rathinam and Chan, 2018). In addition, increasing evidence indicates that in chronic non-infectious diseases, such as diabetes and its complications including DKD and atherosclerosis, persistent systemic or local inflammation plays a critical role in the progression of disease (Pichler et al., 2017; Tang and Yiu, 2020; Engelen et al., 2022; Rohm et al., 2022). A large number of infiltrated macrophages were observed in the renal biopsy specimens in patients with DKD, and the expression of inflammatory cytokines such as Interleukin 6 (IL-6), Interleukin 1 beta (IL-1β), and tumor necrosis factor alpha (TNF-α) were increased (Navarro-Gonzalez et al., 2011; Araujo et al., 2020). In recent years, the microarray analysis has also showed that the expression of pro-inflammatory genes and fibrosis-related genes were significantly increased in patients with DKD or animal models, which was accompanied with increased levels of inflammatory cytokines in peripheral blood specimens (Wilson et al., 2019; Levin et al., 2020; Sur et al., 2021). These data further indicate that there is a notable association between inflammation and the development of DKD.

In this review, we discuss the role of some novel and key pro-inflammatory signaling pathways in the progression of DKD, such as the nuclear factor kappa B (NF-κB) signaling pathway, toll-like receptors (TLRs) and myeloid differentiation primary response 88 (TLRs/MyD88) signaling pathway, adenosine 5′-monophosphate-activated protein kinase (AMPK) signaling pathways, inflammasome activation, mitochondrial DNA (mtDNA) release and hypoxia-inducible factor-1(HIF) signaling pathway. We also introduce the role of anti-hyperglycemic drugs, non-steroidal selective MRA and traditional Chinese medicine in anti-inflammation during the treatment of DKD.

## Hyperglycemia-Induced Inflammation in DKD

Hyperglycemia and insulin resistance are the most common pathological characteristics in patients with DKD and can lead to systemic low-grade inflammation (Saraheimo et al., 2003; Leehey, 2020). Hyperglycemia can lead to overproduction of ROS and mitochondrial DNA release, which further cause inflammation in renal parenchymal cells by different signaling pathways (Sun et al., 2022).

### NF-κB Signaling

The transcription factor NF-κB is a key mediator of inflammation and NF-κB remains inactive while binding to the proteins of the IkappaB (IκB) family (mainly IκBα) (Sun, 2011, 2017; Zhang Q. et al., 2017). There are two pathways for the activation of NF-κB: the canonical pathway and the non-canonical pathway (Zhang S. et al., 2017). Under the stimulation of various immune receptors, such as pattern recognition receptors (PRR), TNF receptors (TNFR), T cell receptors (TCR) and B-cell receptors, intracellular IκBα is degraded and the canonical NF-κB pathway is activated (Sun, 2017). The non-canonical NF-κB activation is primarily associated with ligands of a subset of TNFR superfamily members, such as the lymphotoxin beta receptor (LTβR), B-cell activating factor receptor (BAFFR), Cluster of differentiation 40 (CD40), and Receptor activator of nuclear factor κ B (RANK) (Sun, 2017; Hou et al., 2018). NF-κB mediates the activation of downstream signaling pathways including inflammasome activation, HIF signaling pathways and AMPK signaling pathways (Rius et al., 2008; Afonina et al., 2017). In addition, the activation of NF-kB signaling pathway inhibits the anti-inflammatory pathway, Sirtuin 1 (SIRT1) signaling pathway, which is closely related to the development of non-infectious chronic diseases such as atherosclerosis and DKD (Stein et al., 2010; Kauppinen et al., 2013; Sun et al., 2021). Overall, the NF-kB signaling pathway plays a key role in the inflammatory response.

It has been observed that activated NF-κB signaling pathway is associated with inflammation and fibrosis in DKD (Mezzano et al., 2004; Schmid et al., 2006; Liu P. et al., 2014). Foresto-Neto et al. found that the activation of NF-κB signaling pathway increased the expression of the inflammatory cytokines IL-6, which preceded the kidney damage in the db/db mice (Foresto-Neto et al., 2020). Microarray analysis of renal biopsies in patients with DKD also suggested that a number of NF-κB targets were significantly upregulated (Schmid et al., 2006). These data suggest the key role of abnormal activation of NF-κB in the progression of DKD. A recent study found that delivery of Smad7 siRNA to the kidney through ultrasound microbubbles could reduce the production of IL-1β and monocyte chemoattractant protein 1 (MCP1) in the renal tissue of db/db mice by inhibiting NF-κB signaling pathway (Ka et al., 2012). Sun et al. demonstrated that dephosphorylated P65 NF-κB could attenuate renal inflammation-related damage in STZ-induced diabetic mice by downregulating the expression levels of TNF-α, IL-1β, and cyclooxygenase 2 (COX-2). (Zhang M. et al., 2019). These data further confirm that the NF-κB signaling pathway plays a critical role in the development of DKD and targeting NF-κB signaling pathway might be a potential therapeutic strategy for DKD.

### TLRs-MyD88 Signaling Pathway

Toll-like receptors (TLRs) is an evolutionarily ancient family of pattern recognition receptors and an important component of innate immunity (Tang and Yiu, 2020). Activation of TLRs in response to pathogen-associated molecular patterns (PAMPs) or danger-associated molecular patterns (DAMPs) could lead to inflammation in injured tissue (Fitzgerald and Kagan, 2020; Tang and Yiu, 2020). Myeloid differentiation factor 88 (MyD88) is a critical adaptor protein in innate immunity and central hub in inflammatory response (Xu et al., 2000; Deguine and Barton, 2014), which could respond to the activation of TLRs. The activation of TLRs-MyD88 signaling pathway might initiate the translocation of NF-κB into the nucleus, thus promoting the transcription of pro-inflammatory factors and chemokines, such as IL-1β, IL-6, MCP-1 etc. (Warner and Nunez, 2013; Fitzgerald and Kagan, 2020). These studies suggest that TLRs-MyD88 signaling pathway plays a key role in the inflammatory response.

It has been demonstrated that the high glucose (HG) could activate TLRs-MyD88 signaling pathway in the kidneys of DKD, which is the initiator of renal interstitial fibrosis (Lin and Tang, 2014; Liu and Zen, 2021). Liu et al. found that the mRNA and protein levels of TLR4 and MyD88 increased in renal tubular epithelial cells treated with HG, which was accompanied with the activation of NF-κB and increased expression of chemokine MCP-1 (Liu R. et al., 2014). Zhang et al. found that pharmacological inhibition of MyD88 by LM8, a new small-molecule inhibitor of MyD88, could alleviate the progression of renal fibrosis by inhibiting the activation of NF-kB and reducing the expression of TNF-α and IL-1β in both streptozotocin (STZ)-induced diabetic mice and db/db mice (Zhang et al., 2022). These data indicate that targeting TLRs-MyD88 might be a potential therapeutic option to reduce renal fibrosis in DKD. In addition, there are a series of Chinese herbal medicines to reduce renal fibrosis by inhibiting TLRs-MyD88 signaling-mediated inflammation (Lu et al., 2019; Arigela et al., 2021; Guo et al., 2021).

### Inflammasome Activation

Inflammasome was first proposed by Tschopp et al., in 2002, which could modulate inflammation by the activation of caspases (Martinon et al., 2002; Schroder and Tschopp, 2010; Guo et al., 2015). There are canonical inflammasomes and non-canonical inflammasomes. The former are caspase-1-dependent inflammasomes and the non-canonical inflammasomes depend on caspase-11 in mouse and caspase-4 or caspase-5 (the homologue of caspase-11) in humans (Stowe et al., 2015; Van Opdenbosch and Lamkanfi, 2019). It is well known that the activation of the inflammasome is associated with a series of pattern recognition receptors (PRRs) in the membrane and cytoplasm, which include nucleotide-binding oligomerization domain (NOD)-like receptors (NLRs), Toll-like receptors (TLRs), C-type lectin receptors (CLRs), Rig-I-like receptors (RLRs), and absent in melanoma 2 (AIM2)-like receptors (ALRs) (Schroder and Tschopp, 2010; Strowig et al., 2012; Evavold and Kagan, 2019). This series of PRRs could rapidly respond to danger signals such as PAMPs or DAMPs to activate inflammasomes (Guo et al., 2015).

Accumulated data suggests that the inflammasome contributes to the progression of DKD (Strowig et al., 2012; Masood et al., 2015). Upregulated expression of IL-1β and Interleukin 18 (IL-18) was observed in the peripheral blood or renal tissue of DKD patients (Uzu et al., 2011; Lei et al., 2019). In addition, the activation of the NLRP3 inflammasome was not only present in the renal infiltrating macrophages (Shahzad et al., 2015; Zhang J. et al., 2021), but also in podocytes (Xiong et al., 2020; Wu et al., 2021), tubular epithelial cells (Han et al., 2019; Xie et al., 2019) and mesangial cells (Yi et al., 2017; Tung et al., 2018). Inhibition or knockout of NLRP3 has also been shown to be effective in delaying the progression of DKD. It has been reported that the NLRP3-specific small molecule inhibitor MCC950 could alleviate renal damage and fibrosis in db/db mice (Wang et al., 2017; Zhang W. et al., 2019; Ding et al., 2021) and knockdown of NLRP3 has a significant reno-protective effect in STZ-induced diabetic mice (Wu et al., 2018). In addition to the NLRP3 inflammasome, NLRP1 and NLRC4 inflammasome were also activated in the kidneys of DKD mice and associated with increased urinary albumin excretion and renal damage (Yuan et al., 2016; Soares et al., 2018). Furthermore, Luan et al. demonstrated that NLRC5 gene deficiency also reduced the inflammation and renal damage and delayed the progression of renal fibrosis in STZ-induced diabetic mice (Luan et al., 2018).

Other novel inflammasomes such as NLRP2, NLRP6, NLRP10, NLRP12, and AIM2 are also found to be involved in the regulation of inflammation and their expressions are upregulated in various kidney diseases (Yuan et al., 2016; Luan et al., 2018; Wu et al., 2018). It has been shown that the above inflammasomes participate in the pathogenesis of non-diabetic kidney diseases (Lech et al., 2010; Komada et al., 2018; Valino-Rivas et al., 2020). Valiño-Rivas et al. found that NLRP6 deficiency aggravated ischemia-reperfusion (I/R) induced acute kidney injury (Valino-Rivas et al., 2020). The AIM2 inflammasome is a sensor for endogenous Double Stranded DNA (dsDNA) that mediates canonical and non-canonical inflammasome activation. The expression level of AIM2 inflammasome is also associated with the progression of hepatitis B-related glomerulonephritis and lupus nephritis (Zhang et al., 2013; Zhen et al., 2014; Komada et al., 2018). These data suggested that the inflammasomes might also have potential role in the pathogenesis of chronic kidney disease (CKD). However, there is currently a lack of studies on investigating the effect of inflammasomes above in DKD, thus it needs to be further explored in future.

### AMPK Signaling Pathways

AMP-activated protein kinase (AMPK) is a serine/threonine kinase that is involved in regulating cellular metabolism by activating energy-producing pathways (Cordero et al., 2018; Lyons and Roche, 2018; Larabi et al., 2020) (Wang et al., 2018), In addition, AMPK plays a key role in regulating autophagy (Li and Chen, 2019) and other biological functions. Recent studies have also suggested that AMPK is involved in inflammation, particularly the activation of NLRP3 inflammasome (Youm et al., 2015; Xian et al., 2021). It is wellknown that the AMPK dysfunction is associated with a variety of diseases including diabetes mellitus (Chen et al., 2021) and cancers (Wang et al., 2016).

Interestingly, AMPK signaling pathway was related to the renal inflammation (Garcia and Shaw, 2017). Previous study by our group showed that DsbA-L could inhibit the activation of NLRP3 by activating AMPK phosphorylation in the kidney of DKD mice, while the expression levels of IL-18 and IL-1β were increased in that of DKD mice treated with compound C (an AMPK inhibitor) (Yang et al., 2021a). AMPK also inhibits the expression of inducible nitric oxide synthase (iNOS), thereby inhibiting the activation of NLRP3 inflammasome (Pilon et al., 2004; Liao et al., 2021). These studies confirmed the reno-protective and anti-inflammatory effects of AMPK signaling pathway in DKD. In addition, AMPK activation induced mitophagy, which could clear damaged mitochondria in cells and reduce the production of ROS and activation of NLRP3 inflammasome (Seabright et al., 2020; Yang et al., 2021b; Drake et al., 2021). What’s more, a variety of drugs for the treatments of DKD such as metformin, could also activate phosphorylation of AMPK to inhibit renal inflammation (Zhang X.-X. et al., 2020). Therefore, understanding mechanisms of AMPK signaling pathway would be critical to improving the strategy of DKD prevention and treatment.

### Mitochondrial DNA Release

**FIGURE 1:**
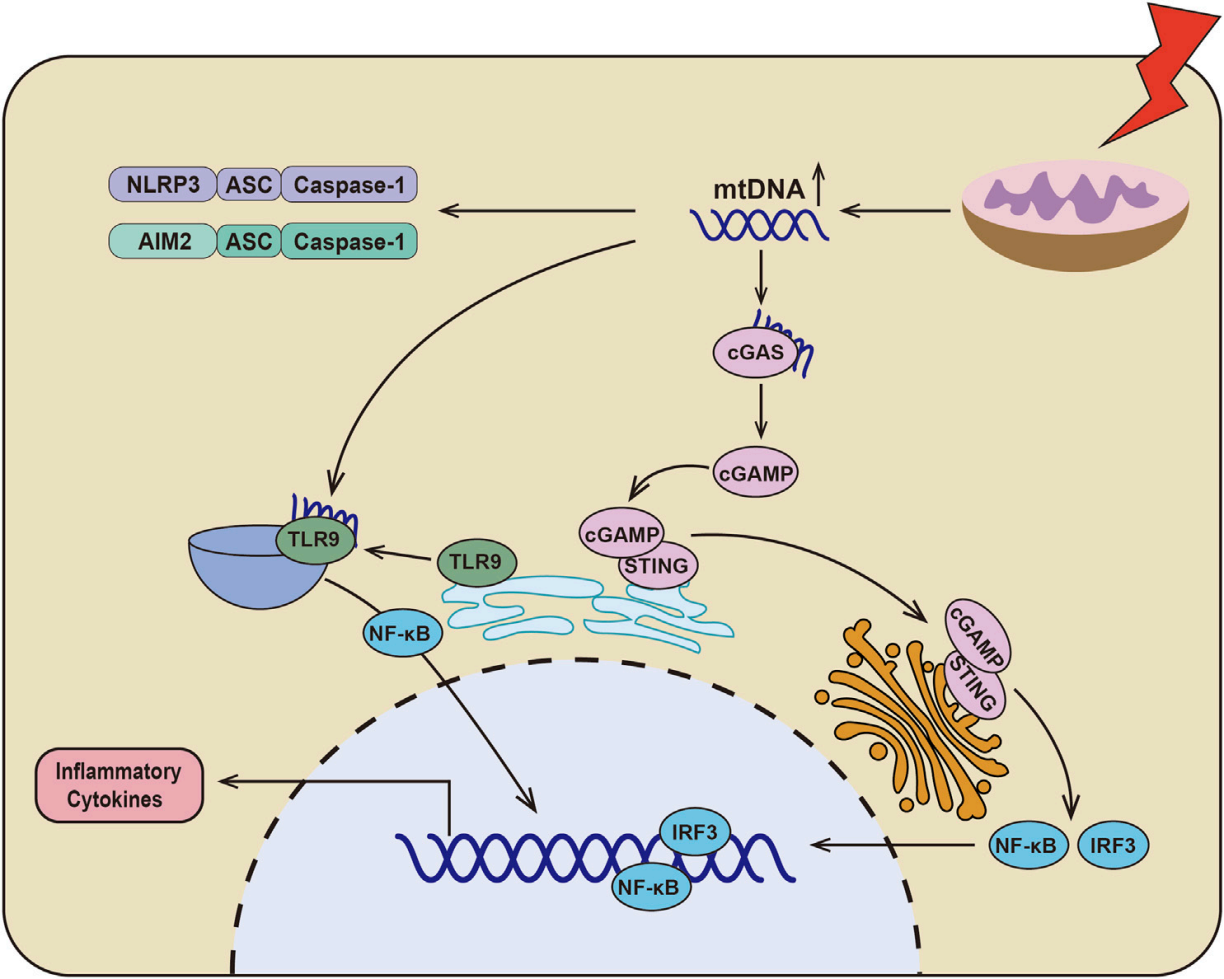
Schematic depicting the relationship between dysfunctional mitochondria and inflammation. In DKD, there are serious mitochondrial dysfunction and mtDNA leakage, which could directly activate NLRP3 inflammasome and AIM2 inflammasome. Additionally, mtDNA could be sensed by cGAS to catalyze ATP and GTP into the second messenger cyclic GMP-AMP (cGAMP). And then cGAMP binds to STING and translocation of the Golgi apparatus to initiate IRF3 and canonical NF-κB signaling pathway. Furthermore, mtDNA activates TLR9/NF-κB signaling pathway by binding to TLR9, which results in the production of inflammatory cytokines.

When mitochondria are dysfunctional, the mitochondrial DNA (mtDNA) translocates to cytoplasm and initiates the inflammation through various signaling pathways (Figure 1) (Zhang et al., 2010; Fang et al., 2016; Riley and Tait, 2020). First, mtDNA, as an endogenous DAMPs, could directly activate the inflammasomes (Nakahira et al., 2011), which conclude NLRP3 (Shimada et al., 2012; Zhong et al., 2018) and AIM2 (Bae et al., 2019; Xu et al., 2021). In addition, mtDNA could initiate the cGAS-STING signaling pathway. The mtDNA is translocated to the cytoplasm and associated with the DNA receptor cyclic GMP-AMP Synthase (cGAS), causing the activation of cGAS, which catalyzes ATP and GTP into the second messenger, cyclic GMP-AMP (cGAMP) (Wu et al., 2013). Stimulator of interferon genes (STING) was localized in the endoplasmic reticulum and activated by cGAMP. STING then translocated to the Golgi apparatus to initiate interferon regulatory factor 3 (IRF3) and canonical NF-κB signaling pathway (Ishikawa and Barber, 2008; Hopfner and Hornung, 2020). Recently, it has also been found that the hypomethylated mtDNA is the ligands of TLR9 for regulating inflammation (Goulopoulou et al., 2012; Rodriguez-Nuevo et al., 2018). When cells are under stress, TLR9 is translocated to endo-lysosome from endoplasmic reticulum (Latz et al., 2004; Leifer et al., 2004; Fang et al., 2016), where hypomethylated mtDNA binds to TLR9 to activate TLR9/NF-κB and TLR9/MyD88 signaling pathways, finally resulting in increased production of TNF-α, IL-6, IL-1β, and MCP-1 (Zhang et al., 2014; Wei et al., 2015; Rodriguez-Nuevo et al., 2018). These data indicat that mtDNA release after mitochondria damage plays a key role in the inflammatory response.

As we know that proximal tubular cells of the kidney are rich in mitochondria and mitochondrial dysfunction and mtDNA release have been recognized in various kidney disease (Forbes and Thorburn, 2018; Ahmad et al., 2021). A large amount of mtDNA was detected in the blood and urine of patients with DKD, which is also related to the severity of interstitial fibrosis (Malik et al., 2009; Huang Y. et al., 2020; Jin et al., 2021). The level of mtDNA was increased in primary mesangial cells treated with high glucose, which is accompanied with enhanced TLR9 activation (Czajka et al., 2015). Chung et al. confirmed that STING gene deficiency significantly reduced renal fibrosis and the expression of TNF-α, IL-1β, IL-6 and MCP1 (Chung et al., 2019). There is no doubt that the activation of the mtDNA-cGAS-STING signaling pathway is related to renal fibrosis in DKD. Meanwhile, Myakala et al. also found that sacubitril/valsartan could regulate mitochondrial function and alleviate mtDNA release to inhibit mtDNA-cGAS-STING signaling pathway, ultimately delaying the progression of DKD. This data indicated that targeting mtDNA may be a powerful solution for the treatment of DKD. In addition, it has been reported that mitophagy inhibits the activation of NLRP3 inflammasome and the secretion of inflammatory cytokines such as IL-1β in DKD (Han et al., 2021). These data suggest that mitochondrial homeostasis disruption and mtDNA release are important initiator in renal inflammation, and it might be a potential therapeutic target for the treatment of DKD.

### HIF Signaling Pathways

Hypoxia-inducible factor (HIF) is conserved in biological evolution and is an important mediator of inflammation (Palazon et al., 2014; McGettrick and O'Neill, 2020). The structure of HIF contains α and β subunits, which include three α subunits (HIF-1α, HIF-2α, HIF-3α) and three β subunits (HIF-1β, HIF-2β, HIF- 3β). It has been reported that HIF-1α and HIF-2α are related to the inflammation (Keith et al., 2011). A large amount of evidence shows that the activation of HIF-1α under hypoxia regulates the transcription level of NF-κB (Palazon et al., 2014). Additionally, the fatty acid oxidation (FAO)-mediated NLRP3 inflammasome is activated when the HIF-2α is deficient in macrophage (Li et al., 2021). Of note, inflammation also results in the activation of HIF signaling pathway (Watts and Walmsley, 2019; McGettrick and O'Neill, 2020). Zhang et al. found that SARS-CoV-2 infection induced nuclear translocation of NF-κB to upregulate the mRNA level of HIF-1α via IL18/IL18R1 (Zhang L. et al., 2021). In addition, endogenous DAMPs could upregulate expression of HIF-1α and induced the production of IL-1β (Tannahill et al., 2013). It could be seen that the HIF signaling pathway and inflammation are interdependent in multiple dimensions.

**FIGURE 2:**
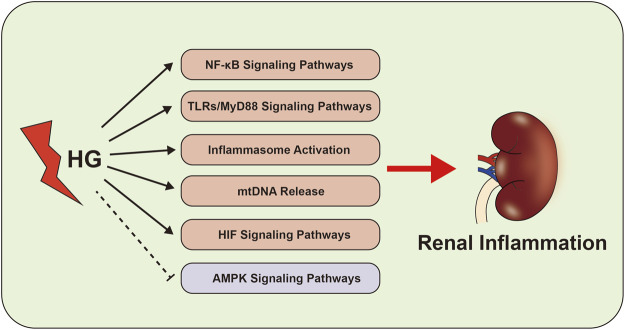
Inflammation-related signaling pathways in the development of DKD. The abnormal of NF-κB signaling pathways, TLRs-MyD88 signaling pathways, inflammasome activation, AMPK signaling pathways, mtDNA and HIF signaling pathway activate the renal inflammation, which cause to kidney damage in DKD.

Recent studies have shown that HIF signaling pathway is closely related to the development of renal interstitial fibrosis in DKD (Li et al., 2019; Jiang et al., 2020). The activation of HIF signaling indicates that the kidney is intolerant to hypoxia, which contributes to the initial adaptive response to hypoxia and tissue repair (Isoe et al., 2010; Garcia-Pastor et al., 2019). Our team has found that HIF-1α activation exerts a reno-protective effect by regulating mitochondrial dynamics through HO-1 in the early stage of DKD in STZ-induced diabetic mice (Jiang et al., 2020). Yu et al. also found that HIF-1α/Parkin/PINK1-mediated mitophagy improved mitochondrial function in renal tubular epithelial cells, whereas inhibition of HIF-1α by YC-1 (a specific inhibitor of HIF-1α) promoted inflammation and increased the production of IL-1β and IL-18 (Yu et al., 2021). These data indicate the reno-protective effect of HIF in the early stage of DKD. However, HIF signaling pathway could also promote chronic inflammation and interstitial fibrosis in CKD including the development of DKD (Wang et al., 2014; Catrina and Zheng, 2021). Co-treatment of mesangial cells with HG and HIF-1α siRNA reduce the production of inflammation and fibrosis factors such as endothelin-1, TGF-β1, CTGF and VEGF (Shao et al., 2016). It has been reported that high doses of MK-8617 (hypoxia-inducible factor-prolyl hydroxylase inhibitor) can promote the tubulointerstitial fibrosis by activating HIF-1α/KLF5/TGF-β1 axis (Li et al., 2019). These data indicate that persistent activation of HIF signaling pathway is an important mediator of the progression of renal interstitial fibrosis and is closely related to the inflammation of DKD (Figure 2).

In addition to be involved in the pathogenesis of DKD, inflammation has recently been shown to play a role in diabetic cardiomyopathy (Quagliariello et al., 2020). Under hyperglycemia condition, it has been demonstrated that the heart would be more susceptible to drug damage or ischemia-reperfusion injury because of hyperglycemia-induced inflammation (Peng et al., 2015; Quagliariello et al., 2020). Quagliariello et al. found that hyperglycemia could activate the NLRP3 inflammasome in cardiomyocytes and enhance the cardiotoxicity of ipilimumab (an anti-cancer drug for breast cancer) (Quagliariello et al., 2020). Additionally, diabetic cardiomyopathy might result in low myocardial reserve (Moir et al., 2006). Treatment of kaempferol to hyperglycemia-induced myocardial damage might reduce ROS production and the expression of TNF-α by inhibiting the NF-κB signaling pathway (Chen et al., 2018). These data suggest that HG leads to cardiac damage and decreases myocardial reserve through the inflammatory signaling pathways.

## Anti-Inflammation Treatment in DKD

The treatments of DKD mainly include management of hyperglycemia, hypertension and hyperlipidemia. Actually, these drugs especially the nonsteroidal selective mineralocorticoid receptor antagonist (MRA) finerenone and some Chinese traditional medicine could alleviate the development of DKD by regulating the inflammation.

### Metformin

Metformin has been used for treatment of diabetes mellitus since 1950s, and is now the most widely used anti-hyperglycemic drug (Flory and Lipska, 2019). There’s a lot of evidence that metformin has not only anti-hyperglycemic properties, but also reno-protective effect. A cohort study showed that treatment of 10,426 patients with type 2 diabetes and chronic kidney disease at stage 3B with metformin significantly reduced the risk of all-cause mortality and ESRD events (Kwon et al., 2020). Similar result was observed in metformin treated DKD animal models, which also showed improved renal function and alleviated renal tubulointerstitial fibrosis (Zhang Q. et al., 2017). Recently, studies have shown that the reno-protective effect of metformin might relate to the mechanisms of anti-inflammation. Among them, AMPK signaling pathway might be the most important anti-inflammatory mechanism of metformin. Recently, Ma et al. found that metformin inhibited v-ATPase activity by inducing the binding of PEN2 to the ATP6AP1 subunit, and then activated lysosomal AMPK (Ma et al., 2022). The work of Han et al. suggested that metformin maintained mitochondrial homeostasis through AMPK-mediated mitophagy and inhibited the activation of NLRP3 inflammasome in DKD (Han et al., 2021). In addition to AMPK signaling pathway, metformin could also regulate renal inflammation by HIF signaling pathway (Takiyama et al., 2011), intestinal flora (Induri et al., 2022), and autophagy (Bharath et al., 2020).

### Finerenone

Nonsteroidal selective MRA is a novel drug with cardio-renal protection. Compared with traditional MRAs such as spironolactone and eplerenone, nonsteroidal selective MRA has significantly improved efficacy and safety. Finerenone (also known as BAY 94-8862) is currently approved by FDA for treatment of DKD. A series of clinical studies confirmed the reno-protective effect of finerenone in DKD. The phase III FIDELIO-DKD trial has shown that finerenone significantly delayed renal failure and eGFR decline. In addition to finerenone, several nonsteroidal MRAs are currently in development (Patel et al., 2021), such as AZD9977 (phase I) (Whittaker et al., 2020), apararenone (phase II) (Wada et al., 2021) and esaxerenone (phase III) (Ito et al., 2020), which might also have reno-protective effect in DKD by regulating inflammation.

It has been well known that overactivation of MR is a key event for chronic inflammation by increasing the recruitment of neutrophil, macrophage and Th1 & Th17 cells and upregulating the expression of pro-inflammatory factors and fibrotic-related factors including TGF-α, endothelin 1, PAI-1, CTGF (Young and Rickard, 2015). There is sufficient evidence to suggest that treatment with MRA including finerenone might alleviate the progression of chronic kidney disease and eliminate the renal inflammation by reducing the expression of pro-inflammatory cytokines, such as MCP-1, TNF-α and Matrix metalloproteinase-12 (MMP-12) (Han et al., 2006; Huang et al., 2014). Martínez et al. found that finerenone regulated the activation of NF-kB signaling pathway through neutrophil gelatinase-associated lipocalin (NGAL) and inhibited the inflammation in cardiac remodeling after myocardial infarction (Martinez-Martinez et al., 2017). Bhuiyan et al. also found that esaxerenone, another nonsteroidal selective, significantly reduced the expression of MCP-1 and inflammatory cell infiltration in DKD by inhibiting ROS (Bhuiyan et al., 2019). These studies suggest that nonsteroidal selective MRA can alleviate the progression of DKD through targeting inflammatory factors.

### SGLT2 Inhibitors

Sodium-glucose cotransporter 2 inhibitor (SGLT2i) is a novel anti-hyperglycemic drug by inhibiting reabsorption of glucose in the kidney (Ferrannini, 2017). Several clinical trials have confirmed that SGLT2i have excellent cardio-renal benefit and low risk of hypoglycemia (Perkovic et al., 2019). Interestingly, the SGLT2i could reduce the tissue low-grade inflammation (Bonnet and Scheen, 2018; Packer, 2020). A retrospective study showed that compared to glimepiride, SGLT2i canagliflozin significantly reduced the expression levels of inflammatory markers and fibrosis markers, such as tumor necrosis factor receptor 1 (TNFR1), matrix metalloproteinase-7 (MMP7), Fibronectin (FN) (Heerspink et al., 2019) and IL-6. The anti-inflammatory mechanism of SGLT2i might relate to HIF-1α in DKD (Bessho et al., 2019). In addition, SGLT2i also can regulate mitochondrial energy metabolism through reducing mitochondrial Ca^2+^ concentration and activating AMPK signaling pathway (Hawley et al., 2016; Baartscheer et al., 2017). Recent study demonstrates that Canagliflozin exerts the anti-inflammatory effect by activating AMPK-Akt-eNOS pathway (Heerspink et al., 2019). Interestingly, it has also been reported that SGLT2i could inhibit inflammasome activation. Kim et al. found that compared to sulfonamide, SGLT2i significantly inhibited the activation of NLRP3 inflammasome and reduced the production of IL-1β (Kim et al., 2020). Birnbaum et al. also found that SGLT2i could inhibit the activation of NLRP3 inflammasome and reduce the mRNA expression of IL-1β, IL-6, TNF-α in BTBR ob/ob mice (Birnbaum et al., 2018). In conclusion, the reno-protective effect of SGLT2i in DKD might be related to anti-inflammation.

### DPP-4 Inhibitor and GLP-1 Receptor Agonist

Glucagon-like peptide 1 (GLP-1) is an incretin hormone that stimulates insulin secretion in response to food intake (Baggio and Drucker, 2007). GLP-1 binds to the GLP-1 receptor in the pancreas and then regulates blood sugar by promoting insulin secretion. The half-life of natural GLP-1 is very short (1–2 min) because it is rapidly degraded by the ubiquitous proteolytic enzyme DPP-4 (Bell et al., 1983; Hansen et al., 1999). Recently, dipeptidyl peptidase-4 inhibitor (DDP-4i) and GLP-1 receptor agonists (GLP1RA) have been widely used in anti-hyperglycemic medications by interfering with GLP-1 expression (McGuire et al., 2019; Shaman et al., 2022). Recent studies demonstrated that DDP-4i and GLP-1RA could not only control blood sugar by regulating the secretion of insulin, but also exert anti-inflammatory and reno-protective effects in DKD (Gangadharan et al., 2016; Coppolino et al., 2018; Shaman et al., 2022). Many studies have suggested that DDP-4i could reduce the secretion of MCP-1 from renal parenchymal cells and inhibit the chemotaxis and activation of mono-macrophages (Ishibashi et al., 2011; Shah et al., 2011; Coppolino et al., 2018). DDP-4i could also inhibit the activity of NF-κB signaling pathway in renal tissue and reduce the expression of TNF-α in STZ-induced diabetic mice (Kodera et al., 2014; Gangadharan et al., 2016). Additionally, supplementation of exendin-4 (a GLP-1 analog) could reduce the secretion of pro-inflammatory factors in the kidney of diabetic mouse, including TNF-α, IL-1β, MCP-1, Intercellular Adhesion Molecule 1 (ICAM-1), etc. and alleviate the progression of DKD (Hendarto et al., 2012; Sancar-Bas et al., 2015). Furthermore, Hasan et al. found that in GLP-1R-deficient mice with 5/6 nephrectomy, linagliptin (a DDP-4 inhibitor) also significantly inhibited the expression of TGF-β, Collagen I, and Phospho-Mothers against decapentaplegic homolog 3 (pSMAD3) and reduced renal fibrosis (Hasan et al., 2019). These studies suggest that DPP-4i and GLP-1RA might have a reno-protective role in DKD through anti-inflammation, which is independent of the anti-hyperglycemia effect. The role of GLP-1 and DDP-4i on anti-inflammation have been confirmed, as well. Zobel el al. found liraglutide treatment (1.8 mg/day) could reduce the gene expression of TNF-α, IL-1β in the peripheral blood mononuclear cells (PBMC) of patients with type 2 diabetes (Zobel et al., 2021). The study of Tremblay et al. also indicated that the treatment with sitagliptin significantly reduced the plasma levels of C-reactive protein (CRP), IL-6, IL-18, secreted phospholipase-A2 (sPLA2), soluble intercellular adhesion molecule-1 and E-selectin (Tremblay et al., 2014).

### Traditional Chinese Medicine

Traditional Chinese medicines (TCM) such as *Astragalus*, Cordyceps sinensis, Tripterygium wilfordii, Fructus arctii, Panax notoginseng, Berberine, etc. are also powerful weapons against DKD (Wen et al., 2017; Zhong et al., 2019). A systematic review and meta-analysis including 29 trials suggested that TCM significantly reduced urinary protein excretion rate and urinary protein (Xiao et al., 2013). Furthermore, Chinese herbal medicines combined with ACEI or ARB have a better effect in reducing urinary protein in patients with DKD compared to ACEI or ARB therapy alone? (Xiao et al., 2013). Some studies indicate that the mechanism of TCM in DKD might be related to anti-inflammation. The study by Zhong et al. suggested that arctigenin, an extract of burdock, reduced proteinuria in diabetic mice through inhibiting NF-κB-mediated inflammation by binding to protein phosphatase 2 A (PP2A) in podocytes exposed to HG ambiance (Zhong et al., 2019). The Tripterygium wilfordii Hook. f (GTW) could inhibit the activation of NF-κB signaling pathway and reduce inflammatory cell infiltration as well as the mRNA expression of IL-1β and TNF-α in STZ-induced diabetic mice combined with unilateral nephrectomy (Wu et al., 2017). Guo et al. found that maackiain could reduce renal inflammation and the expression of MCP-1 and TNF-α by inhibiting TLRs-MyD88 signaling pathway in STZ-induced diabetic rat (Guo et al., 2021). In addition, the anti-inflammatory effect of TCM is also related to reducing ROS, regulating autophagy and inhibiting epithelial-mesenchymal transition (Li et al., 2017; Zhang et al., 2021a; Zhang et al., 2021b). All of these researches demonstrate that TCM might be a potential weapon for the treatment of DKD by anti-inflammation.

In addition to TCM, nutraceuticals derived from Chinese herbal medicines could prevent DKD, for example, boswellic acid, curcumin and quercetin (Lu et al., 2015; Lu et al., 2017; Asgharpour and Alirezaei, 2021). A systematic review and Meta-analysis revealed that curcumin supplementation significantly improved blood sugar, lipid and blood pressure control and decreased the serum creatinine in DKD patients (Jie et al., 2021). Lu et al. also found that curcumin could delay fibrosis progression in DKD mice by inhibiting the initiation of NLRP3 inflammasome and reduce the expression of IL-1β in renal tissue (Lu et al., 2017). Additionally, the present studies have revealed the protective effect of quercetin, a natural AMPK activator, in STZ-induced diabetic rat (Zhang J. et al., 2021). Lu et al. also found that treatment of quercetin could alleviate the progression of renal fibrosis in STZ-induced diabetic rat (Lu et al., 2015).

However, TCM has limitations such as low bioavailability, which greatly limits its broad application in the treatment of DKD (Liu et al., 2013; Zhang C. et al., 2019). The study of Rosso et al. suggested that hyaluronic acid (HA-NPs) and sub-micron particles (a CD44-targeted vector) were effective means to deliver bio-actives to targeted tissue in a specific and controlled manner (Rosso et al., 2013). This suggests that delivering TCM to the kidney might be a potential strategy to improve the bioavailability. Importantly, the expression of CD44 is low in the normal kidney and would specifically increase during renal damage (Zhao et al., 2019). The study of Hu et al. confirmed that CD44-targeted hyaluronic acid-curcumin prodrug could reduce the ROS in renal tubular epithelial cell. Moreover, Wang et al. have found the bioavailability and therapeutic effect of rhein, an active ingredient of TCM, were significantly improved in STZ-induced diabetic mice through kidney-targeted rhein-loaded liponanoparticles (Wang et al., 2019). This study further confirmed the feasibility of kidney-targeted therapies. Thus, it might be effective to transport TCM to the kidney through hyaluronic vector, liponanoparticle, etc. However, this conjecture needs be verified through further experiments.

### Mesenchymal Stem Cells and Extracellular Vesicles

Mesenchymal Stem Cells (MSCs) and extracellular vesicles (EVs) are current research hotspots and pre-clinical agents for the treatment of DKD (Tang et al., 2019; Savio-Silva et al., 2020; Shen et al., 2020). Previous studies suggested that systemic injection of MSCs improved glycemic control but showing no renal protection (Savio-Silva et al., 2020). Recent evidence suggests that proteinuria is decreased after inducing renal homing of MSC by ultrasound-targeted micro-bubble destruction (UTMD) (Marquez-Curtis and Janowska-Wieczorek, 2013). Xiang et al. found that intravenous injection of human umbilical cord-derived MSCs could reduce the expression of inflammatory factors such as IL-6, IL-1β and TNF-α in the kidney and blood, and alleviate the renal interstitial fibrosis in the STZ-induced diabetic rat (Xiang et al., 2020). These studies suggest that MSCs treatment might be beneficial to reduce the renal inflammation and alleviate the progression of DKD.

The EVs include exosomes, microvesicles and apoptotic bodies. It has been found that the EVs play an important role in the progression of DKD (Tang et al., 2019). Under the stimulation of exosomes secreted from HG-treated macrophages, the mRNA and protein levels of TNF-α, IL-1β and MCP-1 in renal mesangial cells increased. Furthermore, intravenous injection of the exosomes into C57BL/6 mice resulted in the increased expression of TNF-α, pro-IL-1β and IL-1β and renal interstitial fibrosis (Zhu et al., 2019). This data suggested that EVs secreted from damaged macrophage or renal cells could promote renal inflammation and interstitial fibrosis in DKD. Additionally, Jiang et al. have found that intravenous injection of the exosomes secreted by human urine-derived stem cells could inhibit the apoptosis of podocytes and renal tubular epithelial cells, and reduce urinary microalbumin excretion in STZ-induced diabetic rat (Jiang et al., 2016). This suggests that EVs derived from MSCs may be excellent biological agents to alleviate the progression of interstitial fibrosis in DKD.

## Discussion

Inflammation plays an important role in the progression of DKD (Perez-Morales et al., 2019). In this review, we have discussed and summarized the role of NF-κB signaling pathways, AMPK signaling pathways, TLRs-MyD88 signaling pathway, inflammasome activation, mtDNA release and HIFsignaling pathway in the inflammation-induced renal damage of DKD. To date, drugs directly targeting inflammation are limited in treatment of patients with DKD, but the commonly used anti-hyperglycemic drugs such as metformin, SGLT2i, DPP-4i, GLP-1AR and TCM also exhibit excellent anti-inflammatory effect. Interestingly, finerenone (a reno-protective nonsteroidal selective MRA) also exhibits strong anti-inflammatory efficacy to protect the kidney and heart in patients with DKD. This data suggested that attention should be paid to inflammation and anti-Inflammation therapy in DKD in future research.

In addition, MSCs and EVs have shown the curative effect and anti-inflammatory effect in the treatment of DKD animal models (Tang et al., 2019). However, both MSCs and EVs are mainly used for animal experiments currently and their efficacy and safety are uncertain in human body.
